# New Endoscopic Devices and Techniques for the Management of Post-Sleeve Gastrectomy Fistula and Gastric Band Migration

**DOI:** 10.3390/jcm13164877

**Published:** 2024-08-18

**Authors:** Carlo Felix Maria Jung, Cecilia Binda, Luigi Tuccillo, Matteo Secco, Giulia Gibiino, Elisa Liverani, Chiara Petraroli, Chiara Coluccio, Carlo Fabbri

**Affiliations:** Gastroenterology and Digestive Endoscopy Unit, Forli-Cesena Hospitals, AUSL Romagna, 47121 Forlì, Italy

**Keywords:** sleeve gastrectomy, fistula, endoscopic treatment, gastric band erosion, endoscopic gastric band removal

## Abstract

Post-sleeve gastrectomy fistulas are a rare but possibly severe life-threatening complication. Besides early reoperation and drainage, endoscopy is the main treatment option. According to the clinical setting, endoscopic treatment options comprise stent or clip placement. New endoscopic therapies have recently gained attention, including endoscopic vacuum therapy, VacStent therapy, endoscopic internal drainage with pigtail stents, endoscopic suturing and stem cell injection. In this narrative review, we shed light on recent literature, developments, indications and contraindications of these treatments. Intragastric gastric band migration is a rare complication after gastric band positioning. Reoperation can sometimes be difficult, especially when a gastric band has already migrated far into the stomach. Endoscopic retrieval can be a valid, non-invasive therapeutic solution. We reviewed the current literature on this matter.

## 1. Introduction

Sleeve gastrectomy is the most performed surgical intervention for morbid obesity in the world, with an estimated annual number of interventions of 160,609 in 2022 in the US [1,2]. According to recent guidelines, sleeve gastrectomy is indicated in grades II and III obesity without comorbidity and in grade I obesity with obesity-related comorbidity [3]. The procedure is not the first choice in patients presenting with clinically important reflux disease as it may enhance symptoms. According to recent literature, 1.6% of patients undergoing sleeve gastrectomy develop post-sleeve leaks or fistulas [4,5]. In the case of gastric sleeve operations, leaks are generally situated alongside the staple line. Staple line leaks are more frequent at the proximal third of the staple line (in about 85% of all cases) and less frequent in the midst or at the distal part [6,7]. In about 80% of all cases, leaks are diagnosed within the first 2 weeks postoperatively [4]. General treatment strategies in literature are reoperation with re-suturing in the first days after intervention if tissue is not friable or otherwise endoscopic treatment.

Various endoscopic techniques and devices exist for endoscopic treatment, including stenting, clipping, suturing and several draining methods. Nevertheless, clear recommendations and guidelines are lacking, as evidence is not supported by randomized controlled studies but mainly by retrospective unicentric data. 

Besides stenting (closure rates up to 92%), over-the-scope clip placement (ca. 67% leak closure rates) has gained significant attention and has become the mainstay of endoscopic leak treatment [5]. Recently, other endoscopic techniques have been developed to broaden the spectrum of endoscopic options. In this short narrative review, we focus on recent devices for endoscopic treatment of staple line leaks after sleeve gastrectomy and their possible future applications. Additionally, we present new endoscopic treatment options for gastric band erosions. 

## 2. Methods

We selected articles discussing the topic of post-sleeve gastrectomy fistula and gastric band erosion, paying specific attention to new devices and techniques available for their management. We developed a non-systematic review article using the following electronic sources: PubMed, EMBASE, Google Scholar, Ovid, MEDLINE, Scopus, the Cochrane controlled trials register and Web of Science. We used the following search terms alone and in combination: “endoscopic devices”, “endoscopic tools”, “endoscopic interventions”, “novel endoscopy techniques”, “post sleeve gastrectomy complications”, “complications after sleeve gastrectomy”, “post-gastric sleeve surgery complications”, “endoscopic treatment”, “endoscopic therapy”, “endoscopic management”, “sleeve gastrectomy”, “gastric sleeve”, “sleeve surgery”, “endoscopic procedures”, “endoscopic innovations”, “advanced endoscopy”, “complications”, “adverse events”, “treatment outcomes”, “new endoscopic devices”, “novel endoscopy tools”, “emerging endoscopic technologies”, “post-gastric sleeve complications”, “complications following sleeve gastrectomy”, “endoscopic interventions”, “endoscopic management”, “endoscopic approaches”, “gastric band erosion”. From this search, 104 articles were identified and subsequently examined by two investigators. They selected articles that reported data related to humans (inclusion criterion) and excluded works without full-text availability, works not in English, book chapters and abstracts and articles published before 1990 (exclusion criteria). The final selection resulted in 91 articles. Finally, we evaluated supplementary references among the articles evaluated in the first search round.

**Figure 1:**
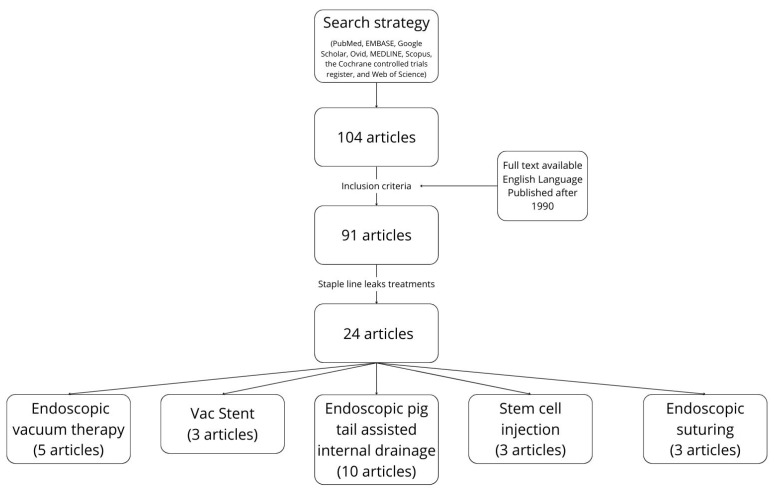
Article identification diagram.

Then, we focused on recent publications discussing the treatment of staple line leaks, including 5 articles about endoscopic vacuum therapy, 3 articles about VacStent placement, 10 articles about internal drainage with pigtail plastic stents, 3 articles about stem cell injection and 3 articles about endoscopic suturing, excluding already well-described techniques such as stent and clip placement (see also Figure 1).

### 2.1. Endoscopic Vacuum-Assisted Closure (EVAC)

Endoscopic vacuum-assisted closure (EVAC) utilizes a sponge inserted endoscopically to absorb fluid, facilitating rapid control of sepsis, reducing the risk of contamination/superinfection and promoting granulation with tissue formation [8]. The sponge can be positioned within the cavity (intracavitally) in cases of accessible leakages with a large opening (≥9 mm) or at the level of the esophageal wall defect (intraluminally) if the orifice is too small. Connected to an external vacuum device through a nasocystic or nasogastric catheter, suction is applied with a continuous negative pressure ranging from −70 to −125 mmHg [9]. This technique promotes granulation tissue formation and fluid extraction, improves blood flow and aids leak closure [9,10]. Additionally, it helps divert acid and bile fluids, which could further damage the leak and lead to inferior wound control. 

Macroporous low-density sponges are preferred for their superior debriding capacity and stronger contraction under negative pressure, leading to significant wound cavity shrinkage (macro-deformation). Permeable films, such as open-pore film drains, offer advantages over polyurethane foam-based drains, depending on clinical indication. They facilitate easier placement due to their smaller diameter and reduced adherence to the wound cavity, thus simplifying removal [11,12]. Open-pore film drains can also be mounted on top of a normal suction tube, creating a smaller device that may be inserted into cavities with a diameter of <10 mm [8].

Sponges are usually changed between 3 and 5 days, depending on the local findings and the clinical course. 60 min for initial sponge placement and 30–60 min for the exchange is the average time considered. For the placement of a new sponge, we always recommend using an overtube to ensure and ease the passage of the upper esophageal sphincter. Sponge removal without exchange takes less than 10 min on average and does not require general anesthesia. Usually, the sponge is removable without any problems. If moderate pulling does not mobilize the sponge, we recommend waiting for a period of 24 h, switching off negative pressure and trying again the next day. The sponge can also be carefully mobilized with endoscopic forceps [10]. 

Thanks to its dual functionalities—sealing the defect and internally draining secretions—endoscopic negative pressure therapy proves versatile even in highly intricate scenarios. As a result, its range of applications surpasses that of any other endoscopic device. Originally, the sponge technique was first applied for lower GI leaks; later, it was transferred to the upper GI. The data on EVT in staple line leaks are based on retrospective studies. Studies published so far are shown in Table 1. Regarding efficacy, Ahrens et al. showed in their retrospective analysis a leak closure rate of 90% after a median treatment time of 17 days for gastric leaks after sleeve gastrectomy and gastric bypass [9]. Leeds et al. reported their experience of EVT therapy in staple line leaks in 2016. Here, nine patients were treated with an average of 10.5 EVT changes over a median therapy treatment period of 50 days, leading to 100% leak closure. In this cohort, six patients had laparoscopic re-exploration/tentative for surgical closure without success. Additionally, five patients were priorly treated with SEMS placement without leak resolution [10]. Another retrospective study by Archid et al. showed an 85.7% efficiency rate of leak closure after 2 (2–10) EVT procedures with a median treatment time of 7.29 ± 7.43 days. In this study, 11 patients were only treated with EVT, 3 with additional laparoscopic drainage and 1 patient with radiologic drainage. One patient in this group had EVT-associated bleeding, and one patient died due to a cause unrelated to the EVT procedure [11]. The most frequent related complications of EVT are stenosis and bleeding, happening in less than 10% of all cases. No randomized controlled trials of EVT in staple line leak closure so far exist. Difficulties that may arise during treatment are due to the size of the leak, which may sometimes be too large for the devices currently available. For patients with hemodynamic instability, surgery is still the first option [12]. Table 1 summarizes the literature on behalf of EVT in sleeve leakages. Supplementary Material S1 provides a video of EVAC treatment (Video S1). 

### 2.2. VacStent

The VacStent (manufactured by VACStent GmbH (Fulda, Germany) and distributed by Microtech-Europe GmbH (Düsseldorf, Germany)) is a new device for the treatment of gastrointestinal leaks and fistulas, combining the advantages of passive leak orifice covertube by a stent and active wound closure by the sponge component. The VacStent was initially proposed for leaks after esophageal resection but was soon also used in patients with sleeve staple line leaks [15,16].

The VacStent was designed to overcome the problem of iatrogenic stenosis induction by vacuum therapy and to allow patients to have oral liquid intake during therapy. The VacStent is inserted into an introducer system that can be deployed endoscopically over the wire, much like other conventional SEMS. This introducer system (with a diameter of 4.2F and a length of 100 cm) contains the VacStent mounted on an inner catheter and encased by an outer tube. Upon retracting the outer tube, the VacStent is freed and expands into a dumbbell shape with an inner diameter of 14 mm. The fanged ends of the VacStent feature a 30 mm lumen, which aligns the sponge cylinder with the intestinal lumen. This facilitates circular EVT along the entire length of the sponge cylinder (50 mm) [15,16]. The VacStent can be left in place for a period of 5 days to develop maximum efficacy. The EVT component helps fix the device along the mucosa. In the VacStent Trial study by Lange et al., an average of 2.7 stent changes was necessary for effective leak healing [15]. Prior to VacStent removal, thorough retrograde rinsing of the sponge via the drainage tube (using at least 40 mL 0.9% NaCl) is advised.

Additionally, suction should be halted for a period of 2 to 4 h before removing the VacStent. Endoscopic removal is carried out using forceps to grasp the retrieval loops positioned at the ends of the VacStent [15,17]. The disadvantages of this device, compared to the EVT only, are the need for a stent change after 5 days, the difficulty of using it in complex wound cavities without wide access to the esophagus and the costs. Obviously, the device cannot be inserted into wound cavities. Otherwise, regarding the leak size, the length of the sponge component limits its applicability. In summary, VacStent has demonstrated itself as an innovative medical tool that combines the advantages of EVT and stenting, offering simplicity and safety in application. It enables prompt closure of wounds and efficient drainage of endoluminal wound cavities, effectively controlling septic focus and promoting accelerated morphological healing. The unobstructed passage through the VacStent facilitates rapid postoperative nutrition and endoscopic access beyond the leak site. With its design, mechanism of action and user-friendly nature, there is a promise that the VacStent could enhance clinical outcomes in complications after esophageal resections, bariatric procedures and perforations [15,18].

Current studies demonstrate high efficacy in staple line leak closure in up to 80–100%, but these results need to be interpreted carefully due to the scarcity of data available so far. Currently, a multicenter observational study (VacStent Registry, Clinical trial number NCT04884334) is collecting data to further clarify the efficacy of the VacStent in patients with spontaneous, iatrogenic or postoperative leakages in the esophagus or colon. Table 2 summarizes studies available regarding the therapeutic effects of VacStent application. Supplementary Material S2 provides a video of the VacStent treatment (Video S2).

### 2.3. Endoscopic Pig Tail-Assisted Internal Drainage 

**Figure 2:**
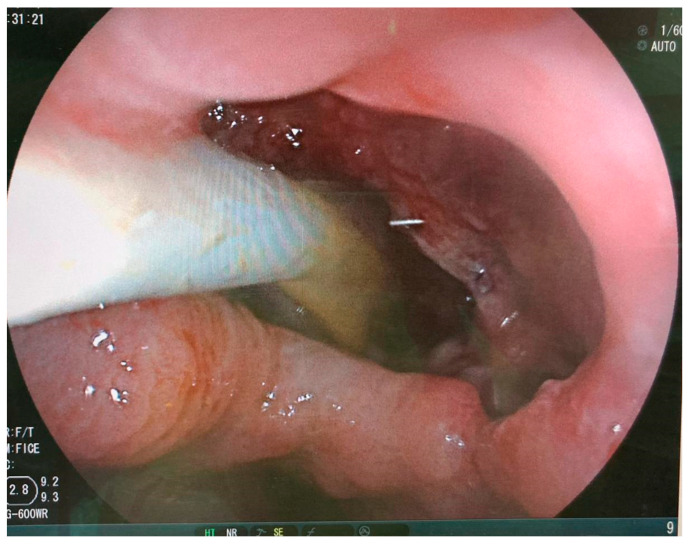
Double-pigtail stent placement across a proximal staple line leak after sleeve gastrectomy.

**Figure 3:**
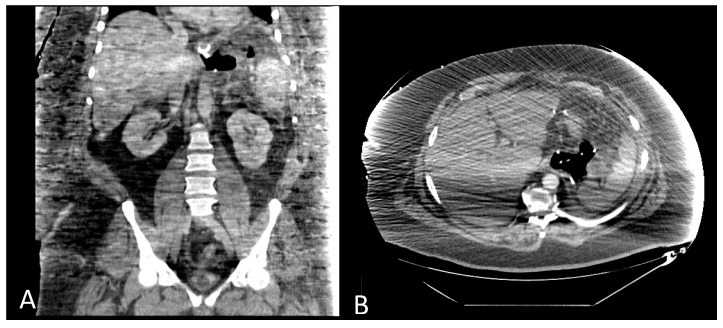
(**A**,**B**) Coronary and transversal slides of a CT scan showing two double-pigtail stents placed into an abdominal cavity between the stomach and spleen.

Treatment with endoscopically inserted double pigtail stents has been recently proposed in the European literature [21,22]. Before any attempt to close the wall defect, the first step is to ensure appropriate drainage of leak cavity content; the endoscopic internal drainage (EID) placed through the leak into the cavity might be the first option for well-circumscribed collections. In addition, when combined with surgical cleansing in patients presenting with severe sepsis, EID allows for the early removal of surgical drainage, preventing chronic fistula tract formation [21,22,23]. The principle of endoscopic internal drainage (EID) is to direct the drainage toward the lumen of the gastrointestinal tract across the leak and, subsequently, closure of the leak itself as the plastic stents slowly promote wound granulation [24,25]. EID pigtail stents can be placed with a normal or therapeutic gastroscope. Sometimes, a duodenoscope is necessary for placement to overcome steep angles. The opening of the leak is then cannulated with an endoscopic retrograde cholangiopancreatography cannula. Leak and cavity size can be confirmed by injection of water-soluble contrast into the cavity. A 0.035-inch guidewire is then passed through the cannula until it loops in the cavity. A double-pigtail biliary stent (7–10 Fr, 4–7 cm) is then placed into the cavity through the leak, leaving the proximal end of the stent in the stomach or distal esophagus (see Figure 2). In some cases, operators chose even smaller and shorter pigtail stents according to leak and cavity size. If necessary, the procedure can be performed repeatedly, with the placement of a second or third double-pigtail stent alongside the first one [24]. Figure 3A,B show a CT scan where EID pigtails are placed in an intraabdominal cavity for proximal staple line leakage. There can be migration of the DPS into the perigastric cavity, either at the time of deployment or later and can usually be managed endoscopically. 

A more significant and potentially life-threatening adverse event is the migration of the DPS into the splenic artery or the spleen, which may require urgent surgical intervention [22,24,26]. Recent reports have shown that EID may not only be more effective than closure management but may also be more cost-effective than placement of fully covered SEMS [24]. Double-pigtail stents are usually removed or changed after three to six weeks, but no clear indications of removal time or time for exchanges exist [27]. The appropriate time interval for stent exchange and the optimal timing to consider EID failure in leak healing and to proceed with other surgical or endoscopic treatments remain to be defined [21]. No clear recommendations on EID for leak size exist either. But logically, the larger the leak, the higher the probability of stent migration. In a recent study, EID was applied with success even in leaks (after Ivor Lewis resection) with a size up to 20 mm [28]. Laopeamthong and Giuliani et al. report in their latest meta-analysis an overall efficacy of 84.7–91.6% of EID in staple line leaks after sleeve gastrectomy [29,30]. Table 3 shows different studies of EID treatment in staple line leaks after sleeve gastrectomy. 

### 2.4. Mesenchymal Stem Cells and Platelet-Rich Plasma (PLP) Therapy

Among the novel approaches accessible to manage post-sleeve gastrectomy complications, especially Mesenchymal Stem Cell (MSC) injection and platelet-rich plasma (PLP) have emerged over the past two decades, signifying a potential paradigm shift in treating post-sleeve fistulas [39]. MSCs are adhesive cells exhibiting a fibroblast-like phenotype with reservoir function as stem cells for adipocytes, osteoblasts and chondrocytes; when prompted by pro-inflammatory cytokines, MSCs secrete various immunosuppressive molecules, thereby reducing overall inflammation. Additionally, MSCs can foster wound healing and tissue regeneration by secreting TGF-β and fibroblast growth factor, and they can also differentiate into fibroblasts or endothelial cells to form granulation tissue. Hence, MSCs integrate anti-inflammatory and regenerative traits, which are valuable in addressing leaks and fistulas [23]. In 2020, Trevisonni et al. revised the evidence-based data collected concerning endoscopic local delivery of adipose tissue/MSC, showing that this method represents a moderately invasive but relatively safe treatment option to promote leak healing [40].

Most of this data came from single case reports or series of case studies; in reality, the development of larger, multicenter clinical trials faces challenges due to the limited pool of eligible participants and the scarcity of clinical facilities equipped with the necessary expertise and resources for implementing innovations in this domain.

Porziella and Nachira et al. recently showed their results in treating patients with esophageal leaks with harvested emulsified stromal vascular fraction tissue (tSVFem). Here, the subcutaneous tissue of the patient needs to be harvested, mechanically emulsified by passing it through various filters and finally centrifuged. Here, the authors obtained tSVFem, which also included mesenchymal stem cells with extracellular matrix components. Then, this cellular mix was injected in every quadrant around the leak (mean leak size 8 mm, range 4–15 mm) and led to complete healing in all five cases after 7 days with persistent results after months [41,42].

Regarding the specific procedure for treating post-sleeve gastrectomy fistulas, a peripheral whole blood sample is typically obtained from the patient and centrifuged to procure platelet-rich plasma (PRP). The two solutions are then combined, and the resultant 20 mL of MSC-PRP solution is divided into two parts. The initial 10 mL is injected into four quadrants (equal volume) in the submucosal space surrounding the defect using a first syringe. The second syringe, containing 10 mL, is injected into the wall of the fistula tract, with a maximum depth of 2 mm in the fistula wall. Calcium gluconate is added to this second syringe, facilitating solidification [43,44]. When comparing this technique to the current endoscopic armamentarium for treating fistulas, its potential advantages include cost reduction (as the healing time of the fistula may be shortened, leading to decreased expected costs), fewer side effects and complications, regular visualization of the wound cavity and improved clinical outcomes. This procedure has no specific contraindications; however, it should be noted that the fistula is highly unlikely to subsequently close in the presence of radiological or endoscopic evidence of a stricture or a “spiral-shaped” appearance of the sleeve [39,43].

This approach can be recommended for patients with gastric leaks, irrespective of when the leak occurs, if the patient’s hemodynamic condition is stable, and if their nutritional status is adequate. At the moment, there are only a few published series on the endoscopic administration of autologous MSC-PRP for the treatment of gastric staple line leak after sleeve gastrectomy. These show promising results but have only a small number of cases and no control group [39,45,46]. Possible complications to be considered during this treatment are mostly linked to the PRP and MSC extraction, harvested from the proximal tibia; tibial pain is the most frequent, but tibial hematoma can occur too [46].

Overall efficacy reported in the literature is as high as 100%, but larger studies are needed to define specific indications, possible leak sizes and contraindications for this technique. With its particularly demanding infrastructure, in our opinion, this technique will remain in expert hands and highly specialized centers. 

Table 4 shows different studies, including mesenchymal stem cell treatment for staple line leaks after sleeve gastrectomy. 

### 2.5. Endoscopic Suturing

The role of suturing in patients with staple line leaks after sleeve gastrectomy has been studied less. Only recently have small case studies been presented in the literature, making it difficult to evaluate overall applicability, safety and, finally, indications. 

The Overstitch system (Boston Scientific, Marlborough, MA, USA) can be applied via a mono-channel endoscope or via double-working channel endoscopes. Generally, the maneuverability of the endoscope is slightly better when the “old” Overstitch system with a double-working channel endoscope is used. With the Overstitch system, full-thickness transmural sutures can be applied endoscopically, leading to the full closure of the defect. To improve outcomes, generally, pre-treatment of the mucosal rim with argon plasma coagulation around the leak needs to be performed. 

Cai et al. presented two cases of staple line leaks after sleeve gastrectomy, in which full-thickness suturing was applied. Here, one patient presented with a 2 mm (diagnosed 3 months after the operation) and one with an 8 mm leak (diagnosed 8 days after the operation). The patient with the 8 mm leak received additional stent treatment fixed with the Overstich system. Both patients showed full leak closure on follow-up [47]. Another study by Negm et al. randomized patients with staple line leaks to surgical or endoscopical treatment. The median time-of-leak diagnosis in both groups (surgical and endoscopic) was 6 days. In the endoscopic group, five patients received leak closure with suturing using the Overstitch device and had no recurrent fistula on follow-up. Unfortunately, in this study, leak size in patients treated with Overstitch suturing is not reported [48].

Up until now, no clear recommendations have been drawn from the current literature concerning leak size and applicability of the Overstitch suturing system. Contraindications could include friable tissue and strong angulation of the leak, not permitting direct suturing. When suturing is applied for leak closure, drainage of cavities behind the leak must be ascertained. Table 5 shows the current literature regarding endoscopic suturing treatment in staple line leaks. 

### 2.6. Gastric Band Erosion

Gastric band placement was a popular laparoscopic weight loss operation but is now less performed. One of the reasons for its reduced popularity is inferior long-term efficacy compared to sleeve gastrectomy and complications such as band slippage in 1–22%, band erosion in 1%, port site infection in 1.8%, port displacement 2.5–6% and port leak <1% [50]. 

**Figure 4:**
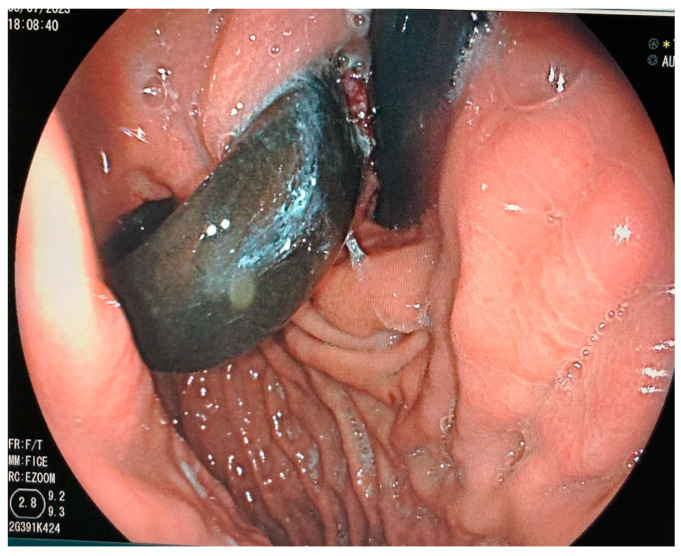
Eroded gastric band (>50%) visualized during routine EGDS.

Gastric band erosion can be managed endoscopically, especially when choosing the right moment. Generally, patients may be asymptomatic, and erosion might be discovered when performing gastroscopy for other reasons, including epigastric pain, melena or suspicion of gastric outlet obstruction. If the gastric band has eroded less than 1/3 of its circumference, early endoscopic removal by cutting the band might not be the best approach in these cases. Some authors have debated the placement of a metal stent into the orifice of the gastric band to induce further erosion [51,52]. Manos et al. promoted stent insertion when a gastric band has eroded <33% of its circumference, a watch and wait strategy when the band has eroded between 33–50%, and a tentative endoscopic removal when the band has eroded more than 50% [52]. Figure 4 shows a case where the gastric band has eroded more than 50% of its circumference. When opting for endoscopic removal, it is important to ensure the reservoir and connecting tube have been removed prior to endoscopy. The technique of endoscopic removal is rather simple. First, a stiff guidewire (450 cm biliary guidewire (Boston Scientific Hydra Jagwire ST, Marlborough, MA, USA) is inserted in the center of the band and passed around. Then, the tip of the Jagwire is externalized by grasping it with pliers and removing the endoscope. Both ends of the Jagwire are then inserted into an external mechanical lithotripter. The endoscope is then inserted again to observe the cutting maneuver when activating the lithotripter. Once the gastric band is cut, it can be moved out of its intragastric wall position with a polypectomy snare. Once completely freed, it can be easily removed trans orally [53]. Normally, gastric wall closure is not necessary, as the fistulized tissue does not have access to the peritoneal cavity. 

The largest cohorts in literature in which endoscopic gastric band removal was performed are presented by Neto, Chisholm, Di Lorenzo and Manos et al. with 82, 50, 50 and 29 patients, respectively (see also Table 6). In the Neto cohort, erosion occurred on an average of 16.3 months after placement, whereas erosion in the Chisholm cohort occurred after 39 months. The time to diagnosis after lap band placement in the Manos cohort was 42 months. Endoscopic removal was successful in 95% of the Neto cohort (78/82 patients), 92% of the Chisholm cohort (46/50 patients), 88% of the Di Lorenzo cohort (44/50 patients) and 93.1% of the Manos cohort. In these cohorts, patients performed endoscopic removal only when the band had migrated at least 50% of its circumference. In case of failure, laparoscopic band removal was performed [52,54,55,56]. 

Failure of endoscopic removal might be due to band cutter failure or connecting tube adhesion [36]. The main complication is the development of pneumoperitoneum [34].

## 3. Discussion

Staple line leaks after sleeve gastrectomy are a possible life-threatening complication. Besides surgical revision, which is still the main intervention strategy in hemodynamically unstable patients, a large variety of endoscopic treatment options exist. SEMS placement for leak closure is the mainstay of endoscopic treatment but may come with pitfalls, including the need for surgical/radiological drainage in case of infected cavities and possible migration. New endoscopic treatment methods include endoscopic vacuum therapy, VacStent therapy, endoscopic internal drainage, stem cell injection or endoscopic suturing. No guidelines exist for these new treatment options, and the choice of devices is in the hands of the applying gastroenterologist/ multidisciplinary team. Local availability, infrastructure and the ability of the endoscopist are key to decision-making regarding whether to choose one method over the other. Where EID, EVT and VacStent therapy might be available to a broad spectrum of gastroenterologists, stem cell injection and endoscopic suturing are less available techniques. They not only need important infrastructure but also experience to be applied. Therefore, these techniques should be reserved for high-volume centers with the presence of a plastic surgeon or, in the case of endoscopic suturing, centers with a lot of experience in endoscopic bariatric suturing therapy.

In view of the current data, no clear conclusion can be drawn on whether the onset of fistula/leak presentation is associated with a certain outcome and whether it affects the choice of a certain endoscopic method. Another very important argument in deciding what kind of endoscopic treatment to prefer is leak size and the presence of an infected cavity behind the leak. EID, for example, is less preferable in patients with leak size >20 mm. Draining methods are preferred in patients with infected cavities. Otherwise, when leak exclusion with SEMS is performed, adequate percutaneous drainage placement is necessary.

During the course of endoscopic treatment, multiparametric reevaluation is constantly necessary (hemodynamic, laboratory and endoscopic), and treatments need to be adapted in case of failure. 

**Figure 5:**
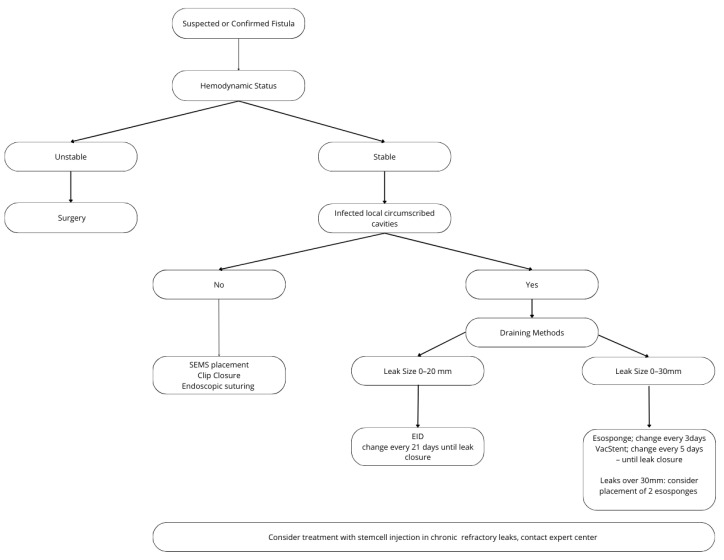
Flow chart for the treatment of post-sleeve gastrectomy leakages.

So far, the data from almost only retrospective, non-comparative publications do not enable the clinician to be guided further. As with this condition, most likely, only retrospective data will exist; it is necessary to examine multicentric data for bariatric and non-bariatric associated leakages to define clearer treatment strategies. At the moment, and taking into account previously proposed algorithms by Spota et al. [57], Chung et al. [58] and Parmer et al. [59], only suggestions could be offered for different indications in different “leak situations” (Figure 5).

The endoscopic treatment method most studied is EID. Many case series and single-center trials exist, reporting excellent outcomes with low clinical treatment impact for the patient, whereas EVT and VacStent therapy must be changed every 3–5 days, double-pigtail stents can be left in place for a long period of time prior to being exchanged, and meanwhile, patients are able to consume food. According to the last studies by Donatelli et al., leak closure rates by EID are as high as 84% and, therefore, comparable to SEMS placement without needing an external drainage tube for draining infected cavities. Another advantage of EID treatment is that endoscopic expertise normally is widely available. 

## 4. Conclusions

New promising therapeutic endoscopic options for the treatment of post-sleeve fistulas are on the rise. Study sizes and design, as well as the lack of comparison between new and standard treatments for now, do not permit drawing early conclusions or treatment preferences. As for leaks after oncologic surgery, multidisciplinary evaluation of the problem is key to defining the best treatment approach. Larger prospective multicenter studies are needed in order to define clear treatment indications for each treatment method. 

## Figures and Tables

**Table 1:** EVT treatment studies in patients with staple line leaks after sleeve gastrectomy.

Authors	Year of Publication	N° of Patients	Technique	Combined	Leakage Diameter in mm	Leakage Site	Success %	Adverse Events
Ahrens et al. [9]	2022	20 (Sleeve + Gastric bypass)	EVT	no	n/a	“Gastric”	90%	no
Leeds et al. [10]	2016	9	EVT	No	n/a	Staple line (6 proximal, 3 proximal and distal)	100%	No
Archid et al. [11]	2021	14	EVT (11 only EVT; 3 combined with laparoscopic drainage; 1 combined with CT drainage	Si	9 < 5 mm; 5 5–10 mm	Staple line (11 proximal, 2 proximal and mid, 1 distal	85.7%	7.1% (bleeding)
Kollmann et al. [13]	2022	8	n/a	n/a	13 mm	n/a	100%	n/a
Mencio et al. [14]	2018	17	EVT	n/a	n/a	n/a	100%	n/a

n/a: not available; EVT: endoscopic vacuum therapy.

**Table 2:** VacStent treatment studies in patients with staple line leaks after sleeve gastrectomy. n/a: not available.

Authors	Year of Publication	N° of Patients	Technique	Combined	Leakage Diameter in mm	Leakage Site	Success %	Adverse Events
Lange et al. [15]	2023	1	First EVT	VacStent (7 changes)	25 × 20 × 30 mm	Esofagogastric junction	100%	VacStent migration
Lange et al. [19]	2023	6	*	*	*	*	80%	Mucosal bleeding, VacStent migration, Aspiration during removal *
La Marca et al. [20]	2024	1		SEMS + VacStent (3 changes) + SEMS	30mm	n/a	100%	

This table demonstrates all studies published so far regarding VacStent therapy for sleeve gastrectomy staple line leaks. * Not specifically mentioned for sleeve gastrectomy patients.

**Table 3:** EID treatment studies in patients with staple line leaks after sleeve gastrectomy.

Authors	Year of Publication	N° of Patients	Technique	Combined	Leakage Diameter in mm	Leakage Site	Bronchial Fistula	% And (n) of Success	Adverse Events
Siddique et al. [24]	2020	20	EID 2 pigtails (95% of pts)	no	n/a	stomach	2	85% (17)	1 migration of EID
Lorenzo et al. [25]	2018	22	EID	no	n/a	stomach	0	86% (19)	none
Benosman et al. [31]	2018	26	EID	SEMS/OTSC	8.3 mm	Staple line	0	88.4% (23)	Stent migration 41%
Deffain et al. [32]	2023	19	EID	Stent/VacStent/OTSC	n/a	Staple line	0	81%	3 migrations of EID
Caiazzo et al. [33]	2020	100	EID	Stent/surgery/radiology	n/a	Staple line	0	90% (90)	None
Donatelli et al. [34]	2021	617	EID		n/a	n/a		84.7%	4.5%
Gonzalez et al. [35]	2018	44	EID	Multiple	>5 mm in 86%	n/a		84%	4.7%
Talbot [36]	2017	7	EID	n/a	n/a	n/a	n/a	100%	n/a
Lazzarin [37]	2020	5	EID	Only EID	n/a	Proximal staple line		100%	
Fuentes Valenzueala et al. [38]	2021	5	EID		<10 mm in 5 patients and 10–20 mm in 4 patients			100%	1/5 bleeding

n/a—not available.

**Table 4:** Mesenchymal stem cell treatment for staple line leaks after sleeve gastrectomy.

Study	Year	Patients (N)	Technique	Leak Dimension	Leak Localization	Fistula	Successful Rate	Complication
Debs et al. [43]	2021	2	MSC-PRP injection only	4–10 mm	Staple line	Yes	100%	None
Moretò et al. [45]	2014	1	Injection of autologous fat	Not specified	Esophagus	Tracheo-esophageal fistula	100%	None
Ben Amor et al. [46]	2023	12	MSC-PRP injection only	Not specified	Staple line	Not specified	100%	4/12 (33.3) Tibial pain or hematoma; epigastric pain, dysphagia tibial hematoma, and one with epigastric pain/dysphagia

**Table 5:** Endoscopic suturing for post-sleeve gastrectomy leaks.

Authors	Year of Publication	N° of Patients	Technique	Combined	Leakage Diameter in mm	Leakage Site	Fistula	% Of Success	Adverse Events
Cai et al. [47]	2014	2	Suturing	1 no, 1 yes (with stent)	2 mm + 8 mm	proximal	0	100%	0
Negm et al. [48]	2023	5	Suturing	suturing	Not reported for the suturing group	proximal	53%	100%	0
Fang et al. [49]	2022	6	EFTR + suturing	no	12 mm	proximal	0	83.3% (5)	0

**Table 6:** Current literature of endoscopic removal of eroded gastric bands.

Name	Year	Number of Patients	Success in %	Procedure Time	Failure n of Patients	Complications
Manos et al. [52]	2023	29	93.1		2	Band buckle trapped in the gastric wall
Neto et al. [54]	2010	82	95	55 min (range 25–150)	4	Pneumoperitoneum in 5 cases.
Chisholm et al. [55]	2011	50	92	46 min (range 17–118 min)	4	
Di Lorenzo et al. [56]	2012	50	88		6	

## Supplementary Materials

The following supporting information can be downloaded at: https://www.mdpi.com/article/10.3390/jcm13164877/s1, Video S1: EVT of a post Ivor-Lewis resection leak; Video S2: VacStent treatment of a post Ivor-Lewis resection leak.

## Abbreviations

DPS	Double-Pigtail Stents
EID	Endoscopic Internal Drainage
EVT	Endoscopic Vacuum Therapy
MSC	Mesenchymal Stem Cells
PLP	Platelet-Rich Plasma
SEMS	Self-Expanding Metal Stent
